# Editorial: Natural Microbial Communities and Their Response to Antibiotic Occurrence in Ecosystems

**DOI:** 10.3389/fmicb.2022.919316

**Published:** 2022-06-02

**Authors:** Anna Barra Caracciolo, Edward Topp, Nikolina Udikovic-Kolic, Paola Grenni

**Affiliations:** ^1^Water Research Institute of the National Research Council, Montelibretti, Italy; ^2^Agriculture and Agri-Food Canada, London, ON, Canada; ^3^Environmental Microbiology and Biotechnology Lab, Rudjer Bošković Institute, Zagreb, Croatia

**Keywords:** microbial functioning, resistome, sub-minimum inhibitory concentration, biodegradation, metal resistance

Environmental contamination with antibiotic residues and antibiotic resistance genes is increasing due to human and animal use of antibiotics. Various anthropogenic sources, such as wastewater treatment plants, effluents and biosolids, reclaimed water, animal manures, and farm runoff, are largely responsible for the diffusion of antibiotic residues and resistant bacteria and genes in soil and water, including drinking water (Barra Caracciolo et al., 2019; Milaković et al., 2020; Rauseo et al., 2021; Zhang et al., 2021; Haenni et al., 2022; Visca et al., 2022; Wilkinson et al., 2022). Microorganisms interact in both intra- and inter-species relationships, modulated by site-specific environmental conditions (e.g., temperature, humidity, multi-contamination), which make each ecosystem unique. Consequently, the effects of antibiotics (including at minimum selective concentrations and at minimum inhibitory concentrations) on natural microbial communities can vary, and is strongly linked to the bacterial resistance and resilience responses (Grenni, 2022). Several ecological aspects regarding the effects (antibiotic resistance spread, biodegradation, microbial functioning alterations) of antibiotics and other co-factors (e.g., heavy metals) on natural microbial communities are reported in this special issue consisting of five original research papers.

The paper by Li et al. reports interesting work on the impact of the broad spectrum antibiotic fosfomycin on wastewater communities (WWC). This antibiotic is an emerging alternative treatment for multidrug-resistant pathogens. Because it is used only when other antibiotics are ineffective, it is very important to monitor the development of resistance against it. The study describes a rapid assessment (with relatively simple tools, combined with metagenomics) of the resistance against fosfomycin that can occur in managed bacterial wastewater communities or in natural microbial communities. Fosfomycin was tested at two concentrations, 10 and 268 mg/L. The latter was considered the 50% maximal effective concentration (EC_50_) for fosfomycin for wastewater microbial communities. They also used the bio-augmented *Pseudomonas putida* KT2440 (a typical member of wastewater treatment plants with inherent resistance to fosfomycin) as a model and marker organism, but it was outcompeted in all experiments. The authors demonstrated that the complex WWCs may tolerate a low fosfomycin concentration, but higher antibiotic presence caused a turnover of community structure and promoted resistance.

Pesce et al. evaluated the chronic effects of sulfamethoxazole (SMX) and sulfamethazine (SMZ) in river sediment microcosms over 28 days. The SMX and SMZ effects on five potential activities involved in C (β-glucosidase, aerobic respiration), N (leucine-aminopeptidase, denitrification), and P (phosphatase) cycles were variable depending on the antibiotic, treatment dose (500 or 5,000 ng/L), and measured activity, with a strong influence of exposure duration. Whereas, the SMZ treatments resulted in only transient effects on the five microbial activities investigated; a significant stimulation of β-glucosidase activity over the 28 days in the communities exposed to the high concentration of SMX was observed. Moreover, a stimulation of aerobic respiration at low SMX concentrations and a reduced concentration at the end of the experiment suggested a potential biodegradation of sulfonamides by sediment microbial communities. The findings suggest that contamination of sediments by sulfonamides is likely to affect biogeochemical cycles, with possible impacts on ecosystem functioning.

Matviichuk et al. measured the concentration of 20 antibiotics and analyzed resistome and microbial taxonomic composition in river biofilms collected upstream (UPS) and downstream (DWS) (at the point of discharge) from a wastewater treatment plant (WWTP). The results showed significant differences between microbiome compositions in biofilms collected UPS and DWS. According to Procrustes analysis, microbial community composition and antibiotics content (sub-minimum inhibitory concentration) may be determinants of antibiotic resistance gene (ARG) composition in samples collected DWS. However, network analysis showed that the occurrence and concentration of antibiotics measured in biofilms did not correlate with the occurrence and abundance of antibiotic resistance genes and mobile genetic elements. The complex matrix of environmental biofilms likely affects the bioavailability and bioaccessibility of antibiotics, which may limit their impact on microbial communities. Specific studies are necessary to better characterize the interactions of biofilm and antibiotics, especially at the microenvironment scale within the biofilm.

Tiwari et al. characterized ARGs and metal resistance genes (MRGs) in different drinking water distribution systems (DWDSs). Two of the waterworks employed artificially recharged groundwater and (ARGW) and used no disinfection in the treatment process. The other three waterworks (two surface and one groundwater source) used UV light and chlorine during the treatment process. Environmental DNA was extracted and then sequenced using the Illumina HiSeq platform for high-throughput shotgun metagenome sequencing. Overall, 430 ARGs were characterized among all samples with the highest diversity of ARGs identified from samples collected from non-disinfected DWDSs. Furthermore, non-disinfected DWDSs contained the highest diversity of bacterial communities. However, samples from DWDSs using disinfectants contained over double the ratio of ARG reads to 16S rRNA gene reads and most of them were MRGs (namely mercury and arsenic resistance genes). The total reads and types of ARGs conferred genes associated with antibiotic groups namely multidrug resistance, and bacitracin, beta-lactam, aminoglycoside, and mercury resistance genes increased in waterworks treating surface water with disinfection.

Werner et al. evaluated the effect of 140 and 154 days of thermophilic composting on the microbial composition of human excreta and sawdust from dry toilets together with straw and green cuttings with and without the addition of biochar. The relative abundance of multiple ARGs, virulence factors, and genes associated with gene-encoding secretion systems (type III and type IV, capable of transferring bacterial proteins into eukaryotic cells; type VI for injecting proteins into other bacteria) decreased during composting. Biochar at the concentration used had very little effect on the efficacy of composting.

Microbial community response to antibiotics is a complex issue and it depends on a combination of site-specific abiotic and biotic factors which make each ecosystem unique. Further studies are desirable for obtaining a more exhaustive overview on this topic.

## Author Contributions

All authors listed have made a substantial, direct, and intellectual contribution to the work and approved it for publication.

## Conflict of Interest

The authors declare that the research was conducted in the absence of any commercial or financial relationships that could be construed as a potential conflict of interest.

## Publisher's Note

All claims expressed in this article are solely those of the authors and do not necessarily represent those of their affiliated organizations, or those of the publisher, the editors and the reviewers. Any product that may be evaluated in this article, or claim that may be made by its manufacturer, is not guaranteed or endorsed by the publisher.
